# Assessment of the predictive value of right ventricular longitudinal strain and functional trends in right ventricular function of patients after LVAD implantation

**DOI:** 10.1007/s10554-026-03780-z

**Published:** 2026-09-17

**Authors:** Carl-Thaddäus Braun, Rene Schramm, Michiel Morshuis, Sabina PW Guenther, Nikolai Hulde, Hermann Körperich, Henrik Fox, Sebastian V. Rojas, Jan Fritz Gummert, Lech Paluszkiewcz

**Affiliations:** 1 Department of Thoracic and Cardiovascular Surgery, Ruhr University Bochum, Heart and Diabetes Center NRW, Bad Oeynhausen, Germany; 2 Heart and Diabetes Center NRW, Institute for Anesthesiology and Pain Therapy, Ruhr University Bochum, Bad Oeynhausen, Germany; 3 Department of Radiology, Nuclear Medicine and Molecular Imaging, Ruhr University Bochum, Heart and Diabetes Center NRW, Bad Oeynhausen, Germany

**Keywords:** Right heart failure, LVAD, Mechanical circulatory support, End-stage heart failure

## Abstract

Right heart failure (RHF) remains a major determinant of morbidity and mortality following left ventricular assist device (LVAD) implantation. Right ventricular free wall strain (RVFWS) and global longitudinal strain (RVGLS) have emerged as promising echocardiographic markers, yet their long-term prognostic utility and suitability to assess changes in RV function remain poorly defined.In this single-center retrospective study, 57 of 95 consecutive HeartMate 3 LVAD recipients (mean age 57 ± 11 years; 79% male) with adequate echocardiographic quality were analyzed. Patients were stratified by RHF phenotype as no RHF (*n* = 28) or RHF (*n* = 29; comprising early acute and early postoperative RHF). RVFWS and RVGLS were measured preoperatively and at 2 weeks, 6 months, 12 months, and 36 months post-implantation using speckle-tracking echocardiography. Predictive value was assessed using logistic regression and ROC analysis; longitudinal change using linear mixed-effects models (LMM); and outcomes using Kaplan-Meier, Cox regression, restricted mean survival time (RMST), and competing-risks analyses.Preoperative RVFWS was lower in patients who developed RHF (-6.0% (IQR-9.5, -4.1) vs. -10.5% (IQR-12.6, -6.8); *p* = 0.037). Preoperative RVFWS predicted RHF with an AUC of 0.66 (95% CI 0.52–0.81), an optimal cut-off of -6.3%, 55% sensitivity and 79% specificity; no other echocardiographic parameter (RVGLS, TAPSE, RVEDD/LVEDD, FAC) discriminated RHF, and no parameter was an independent predictor on multivariable analysis. On LMM, RVFWS declined early in the no-RHF group (Δ + 3.3 at 2 weeks, *p* = 0.005; Δ + 3.0 at 6 months, *p* = 0.022 vs. baseline) and then stabilized; this pattern was robust in complete-case and random-slope sensitivity analyses. RVGLS improved in the RHF group by 12 months (Δ-3.3, *p* = 0.010). No between-group difference was detected at any single time point. One- and two-year survival was 85% and 80% in the no-RHF group versus 61% and 57% in the RHF group; restricted mean survival time to 2 years was 169 days shorter in the RHF group (*p* = 0.018), reflecting significantly higher first-year mortality (HR 3.2; *p* = 0.044) without a later difference. Transplant incidence was similar between groups and competing-risks analysis was concordant.Preoperative RVFWS provides modest discrimination between RHF and non-RHF after LVAD implantation. Serial strain captures dynamic RV changes - an early postoperative decline in RVFWS in patients without RHF and a later recovery of RVGLS in those with RHF - and RHF is associated with worse early survival. Serial RV strain may therefore complement, rather than replace, established risk assessment.

**Supplementary Information:**

The online version contains supplementary material available at https://doi.org/10.1007/s10554-026-03780-z.

## Introduction

The treatment of patients with end-stage heart failure using a left ventricular assist device (LVAD) is continuously advancing. Currently, except for heart transplantation, the HeartMate 3 centrifugal continuous-flow pump is the only treatment option for long-term mechanical circulatory support (MCS) in patients with a failing left ventricle (LV) [1, 2]. Despite the technological advancements in LVAD therapy, right heart failure (RHF) remains a significant complication post-implantation, adversely affecting patient outcomes and survival [3].

RHF during and after LVAD implantation underlies a complex, incompletely understood pathophysiology, with numerous factors contributing to intra- and postoperative right ventricular (RV) dysfunction and failure. Several risk factors were identified and linked to early acute and postoperative RHF. These risk factors range from laboratory parameters such as creatinine to advanced hemodynamic parameters [4].

Several echocardiographic parameters have been evaluated to predict postoperative RV function, such as fractional area change (FAC), tissue doppler imaging-derived tricuspid lateral annular systolic velocity (TASV), RV index of myocardial performance (RIMP), as well as tricuspid annular plane systolic excursion (TAPSE) [5, 6]. However, these measures may not fully capture the complex, dynamic changes in RV performance following LVAD implantation [7].

Global longitudinal strain (GLS) is a promising marker in evaluating myocardial function in patients with heart failure [8, 9]. Recent studies have shown that right ventricular free wall strain (RVFWS) and right ventricular global longitudinal strain (RVGLS) are particularly promising for predicting RHF post-LVAD implantation; however, changes in right ventricular strain during long-term follow-up after LVAD implantation have not been reported [10–12].

This study aims to assess whether preoperative RVFWS and RVGLS predict early acute and early postoperative RHF. Secondly, this study aimed to characterize longitudinal changes in RVFWS and RVGLS over 36 months following LVAD implantation across different RHF phenotypes.

## Materials and methods

### Study Population

**Fig. 1:**
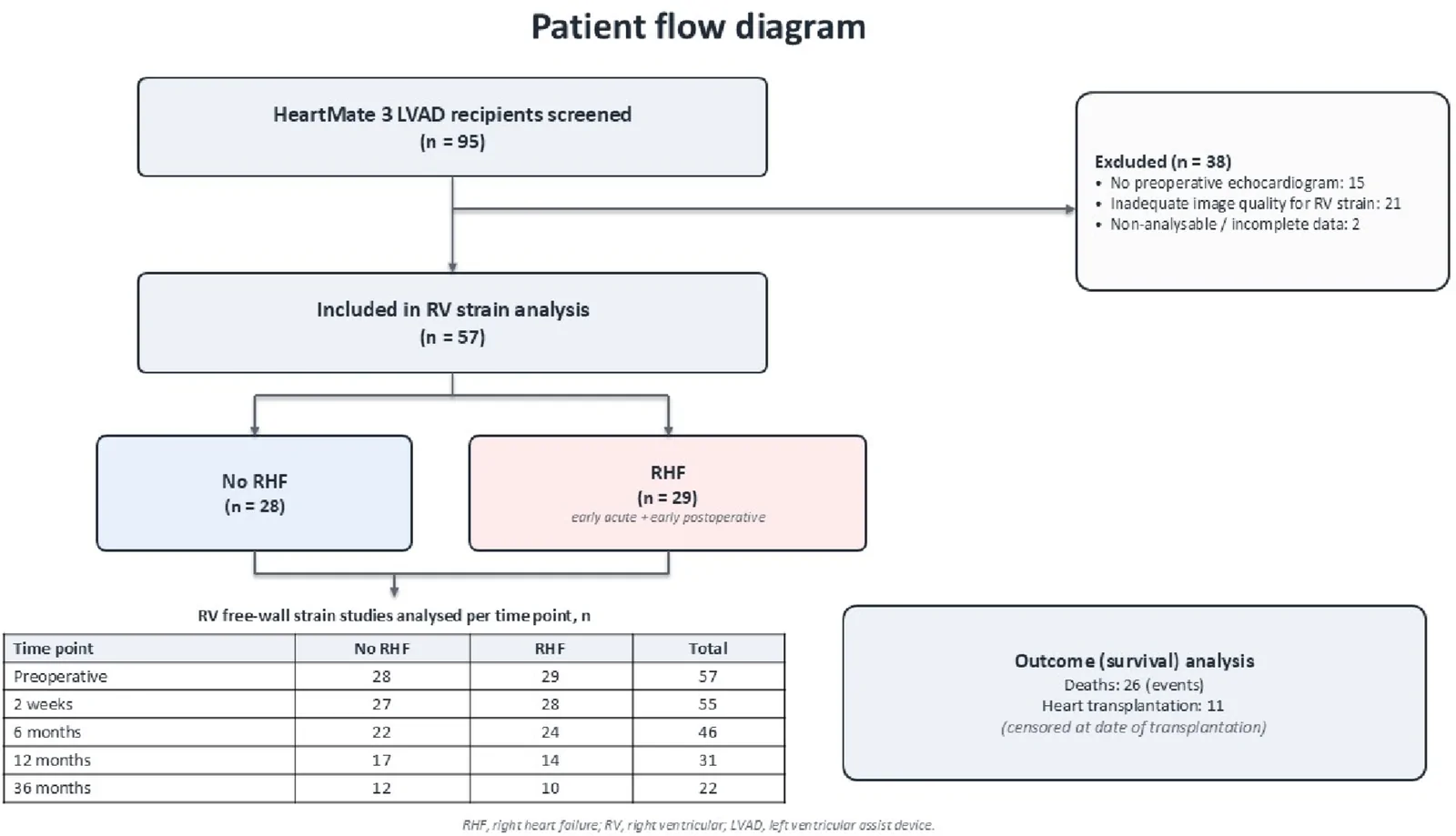
A consort style-flow diagram showing the patients included in the study

We retrospectively analyzed clinical and echocardiographic data from 95 consecutive patients who underwent LVAD (HeartMate 3) implantation at the Heart and Diabetes Center North Rhine-Westphalia between January 2021 and March 2022. Patients with inadequate echocardiographic image quality were excluded. Clinical and laboratory data were obtained using the patient’s electronic medical records. The institutional ethics committee approved the study. A consort style flow-diagram is shown in Fig. 1.

### Right heart failure definition

According to the 2020 Mechanical Circulatory Support – Academic Research Consortium (MCS-ARC) definition, early acute RHF (eaRHF) was defined as patients requiring an RVAD implantation during the operation. Early postoperative RHF (epRHF) is defined as failure to wean from inotropic support within 14 days of the operation or within 30 days following the operation, and at least 2 clinical findings or manifestations listed in the MCS-ARC compendium [13]. Because the individual phenotype subgroups were small and to preserve statistical power, the present analysis compares a single combined RHF group (eaRHF plus epRHF) with patients without RHF; the phenotype composition is reported descriptively.

### Echocardiographic Image Acquisition and Analysis

**Fig. 2:**
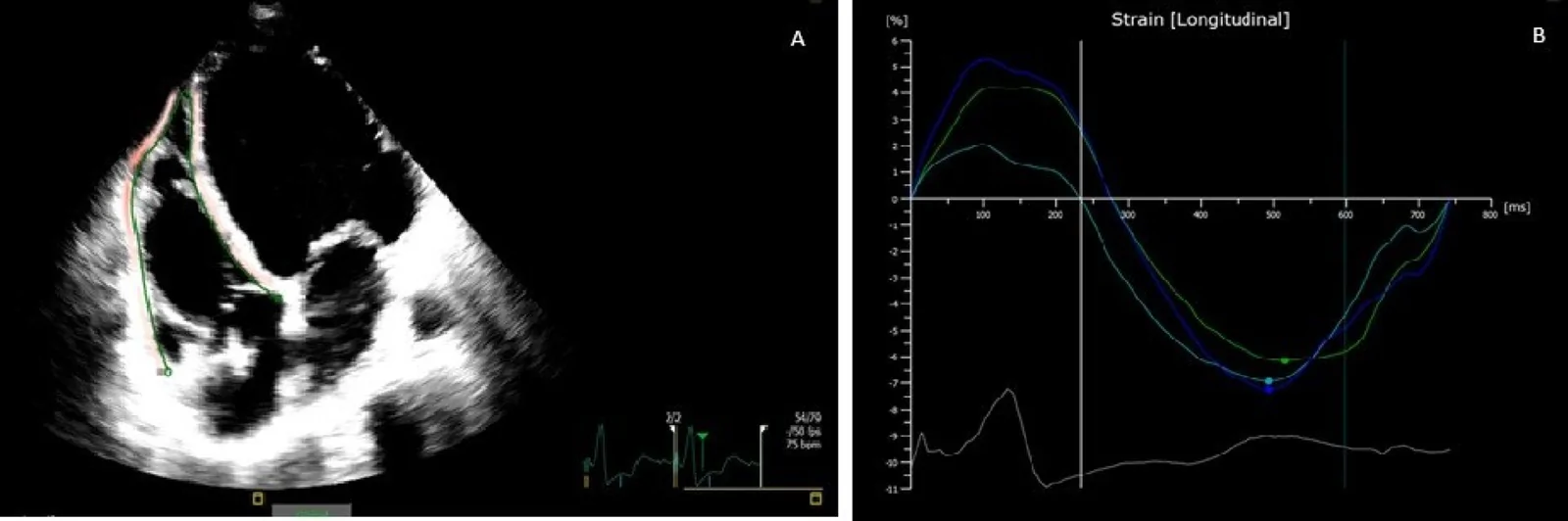
Right Ventricular Longitudinal Strain Analysis in a 64-year old male patient before LVAD implantation. (A) A 4-chamber view was used to analyse the RV-function (B) RV strain curves indicate reduced global RV function, with a peak strain of -7%. The blue, green and purple curves show the regional strain in different areas of the RV.

All images were recorded using the Philips Epiq CVx diagnostic ultrasound system (Koninklijke Philips N.V., Amsterdam, The Netherlands) during routine examinations before and after LVAD implantation. The study included the following echocardiographic measurements: left ventricular end-diastolic diameter (LVEDD), left ventricular ejection fraction (LVEF), TAPSE, right ventricular end-diastolic diameter (RVEDD), the ratio of RVEDD to LVEDD, tricuspid valve insufficiency, as well as RVFWS and RVGLS. Echocardiographic examination was performed routinely during follow-up visits. Patients who underwent heart transplantation during follow-up were excluded from subsequent examinations. Echocardiographic examinations were planned to be assessed at five discrete time intervals: [1] pre-LVAD (within 1 week before the operation) [2], early post-LVAD (within 2 weeks post-LVAD implantation) [3], 6 months post-LVAD implantation [4], 12 months post-LVAD implantation [5], 36 months post-LVAD implantation. Strain analysis was performed offline using the Philips Ultrasound Workspace System (Koninklijke Philips N.V., Amsterdam, The Netherlands) (Fig. 2).

## Statistical Analysis

Continuous variables are reported as mean +/- standard deviation (SD) or median with interquartile range (IQR) according to distribution; normality was assessed with the Shapiro-Wilk test. Groups were compared with the unpaired t-test or Mann-Whitney U test for continuous variables and the chi-squared or Fisher exact test for categorical variables. A two-sided *p* < 0.05 was considered significant.

Longitudinal changes in strain were analyzed using linear mixed-effects models (LMMs; R 4.4.2, packages lme4, lmerTest, and emmeans), specifying strain as a function of group, examination (categorical), and their interaction as fixed effects, with a random intercept per patient. Degrees of freedom were estimated using the Satterthwaite approximation. All available observations contributed under a missing-at-random assumption (no listwise deletion of patients); robustness was examined in complete-case (patients with all five examinations) and random-slope sensitivity models. Estimated marginal means with Tukey-adjusted contrasts were used for within-group change from baseline and between-group differences at each examination. Reported Delta values are estimated marginal mean differences.

The ability of preoperative parameters to predict RHF was assessed using univariable and multivariable logistic regression and ROC analysis, reporting the area under the curve (AUC) with 95% confidence interval, the Youden-optimal cut-off, sensitivity, and specificity; AUCs were compared using the DeLong test.

Survival was analyzed from implantation to death, censoring at transplantation or last follow-up, using Kaplan-Meier estimates, the log-rank test and Cox regression. Because the proportional-hazards assumption (Schoenfeld test) was violated, we additionally report milestone survival, restricted mean survival time (RMST) to 2 years, and hazard ratios stratified by time since diagnosis, before and after 1 year. Death and heart transplantation were also analyzed as competing events (cumulative incidence, Gray test, and Fine-Gray subdistribution model). The minimum detectable between-group difference at 80% power was estimated for the principal strain comparison. Analyses were performed using R (version 4.4.2; The R Foundation for Statistical Computing, Vienna, Austria).

## Results

A total of 95 patients were screened. Of these, 57 patients, with a mean age of 57.3 ± 11.2 years (45 male (79%)), had echocardiographic images of sufficient quality for offline strain analysis. There were 28 patients (49%) in the no RHF group and 29 in the RHF group. Baseline clinical characteristics are summarized in Table 1. Preoperative MCS support was more frequent in the RHF group (13 vs. 2 patients, *p* = 0.002), as was a lower baseline hemoglobin level (10.4 ± 1.5 g/dl vs. 11.8 ± 1.8 g/dl, *p* = 0.002). Patients with RHF were more often classified as INTERMACS 1, whereas patients without RHF were more often classified as INTERMACS 4. More RHF patients had undergone cardiac procedures before LVAD implantation; however, the differences were not statistically significant.

**Table 1:** Baseline clinical characteristics by right heart failure (RHF) status

Parameter	No RHF	RHF*	*p*-Value
	*N* = 28 (49%)^1^	*N* = 29 (51%)^1^	1.000
Age at implantation (years)^2^	56.8 ± 13.2	57.8 ± 9.1	0.740
Male patients^1^	24 (86)	21 (72)	0.674
Height (cm)^2^	177 ± 8	180 ± 8	0.162
Weight (kg)^2^	84.4 ± 16.7	79.3 ± 17.3	0.263
BSA (m^2^)^2^	2.01 ± 0.19	1.98 ± 0.22	0.584
Etiology of HF			
- DCM^1^	19 (68)	21 (72)	0.777
- ICM^1^	9 (32)	8 (28)	0.777
Previous cardiac surgery^1^	4 (14)	11 (40)	0.112
INTERMACS - score			
1^1^	5 (18)	14 (48)	0.084
2^1^	7 (25)	9 (31)	0.706
3^1^	6 (21)	4 (14)	0.529
4^1^	10 (36)	2 (7)	0.032
Destination therapy^1^	8 (29)	9 (31)	0.882
Pre-operative MCS^1^	2 (7)	13 (45)	0.002
Creatinine (mg/dl)^2^	1.7 ± 0.9	1.7 ± 0.8	1.000
Bilirubin (mg/dl)^2^	1.3 ± 0.8	2 ± 1.7	0.053
GPT (U/l)^3^	26 (12–87)	36 (21–82)	0.314
GOT (U/l)^3^	30 (26–79)	47 (29–122)	0.216
Hb (g/dl)^2^	11.8 ± 1.8	10.4 ± 1.5	0.002

^1^number and percentage. ^2^mean and standard deviations, ^3^median and 25th and 75th percentile

Abbreviations: BSA – body surface area, RHF – right heart failure, LVAD – left ventricular assist device, HF – heart failure, DCM – dilated cardiomyopathy, ICM – ischemic cardiomyopathy, INTERMACS – Interagency Registry For Mechanically Assisted Circulatory Support, MCS – mechanical circulatory support, GFR – glomerular filtration rate, GPT- glutamine pyruvate transaminase, GOT – glutamic oxaloacetic transaminase, Hb- haemoglobin, RVAD – right ventricular assist device

*RHF: refers to the patients with both acute right heart failure and early post-implant right heart failure

Postoperatively, the RHF group had a significantly longer duration of catecholamine support (25.5 (20–39) days vs. 6 [4–9] days, *p* < 0.001). The number of additional surgical procedures during LVAD implantation was similar between groups (10 vs. 16 patients, *p* = 0.367). Still, there was a trend toward more re-thoracotomies for bleeding in the RHF group (19 vs. 7 patients; *p* = 0.059). Eighteen patients in the RHF group received RVAD support directly after LVAD implantation. The mean duration of support was 10.1 ± 2.9 days. Postoperative parameters are shown in Table 2.

**Table 2:** Postoperative parameters by right heart failure status

Parameter	No RHF	RHF*	*p*-Value
	*N* = 28 (49%)^1^	*N* = 29 (51%)^1^	1.000
Catecholamine support (days)^2^	6 (4–9)	25.5(20–39)	< 0.001
RVAD support (days)^3^	0	10.1 ± 2.9	0.001
Additional surgical procedures^1^	10 (36%)	16 (55%)	0.367
TVR^1^	4(14)	7 (24)	0.504
MVR^1^	1(4)	1 (3)	1.000
AVR^1^	1 (4)	4 (14)	0.352
ASD closure^1^	4(14)	4 (14)	1.000
Re-Thoracotomy^1^	7	19	0.059

^1^number and percentage. ^2^median and 25th and 75th percentile. ^3^mean and standard deviations

### Echocardiographic Analysis

Preoperative echocardiographic parameters are shown in Table 3. Preoperative RVFWS was significantly less negative in patients who developed RHF − 6.0% (−9.5, −4.1) vs. −10.5% (−12.6, −6.); *p* = 0.037), whereas other parameters did not differ significantly between groups. Strain values were reduced in all patients across all three groups before the operation compared with normal values (Table 3) [14].

**Table 3:** Preoperative Echocardiographic parameters by RHF status

Parameter	No RHF (*n* = 28)	RHF (*n* = 29)	*p*
TAPSE (mm)	16.9 ± 4.4	15.2 ± 3.8	0.147
LVEF (%)	20.5 [18.0, 26.0]	20.0 [15.0, 25.0]	0.200
RVEDD (mm)	46.8 ± 8.6	43.2 ± 11.1	0.178
RVEDD/LVEDD	0.7 ± 0.1	0.7 ± 0.1	0.529
RV-TASV (cm/s)	9.4 ± 3.7	9.0 ± 2.6	0.783
RV FAC (%)	28.8 ± 7.8	25.0 ± 8.5	0.236
RVFWS (%)	−10.5 [−12.6, −6.8]	−6.0 [−9.5, −4.1]	0.037
RVGLS (%)	−7.3 [−9.4, −4.8]	−5.0 [−8.5, −2.4]	0.158

TAPSE – tricuspid annular plane systolic excursion, LVEF – left ventricular ejection fraction, RVEDD – right ventricular end diastolic diameter, LVEDD – left ventricular end diastolic diameter, RVEDD/LVEDD – ratio of right ventricular to left ventricular end-diastolic diameter, RVFWS - right ventricular free wall strain, RVGLS – right ventricular global longitudinal strain, TI – tricuspid valve insufficiency

### RV free wall strain during follow-up

**Fig. 3:**
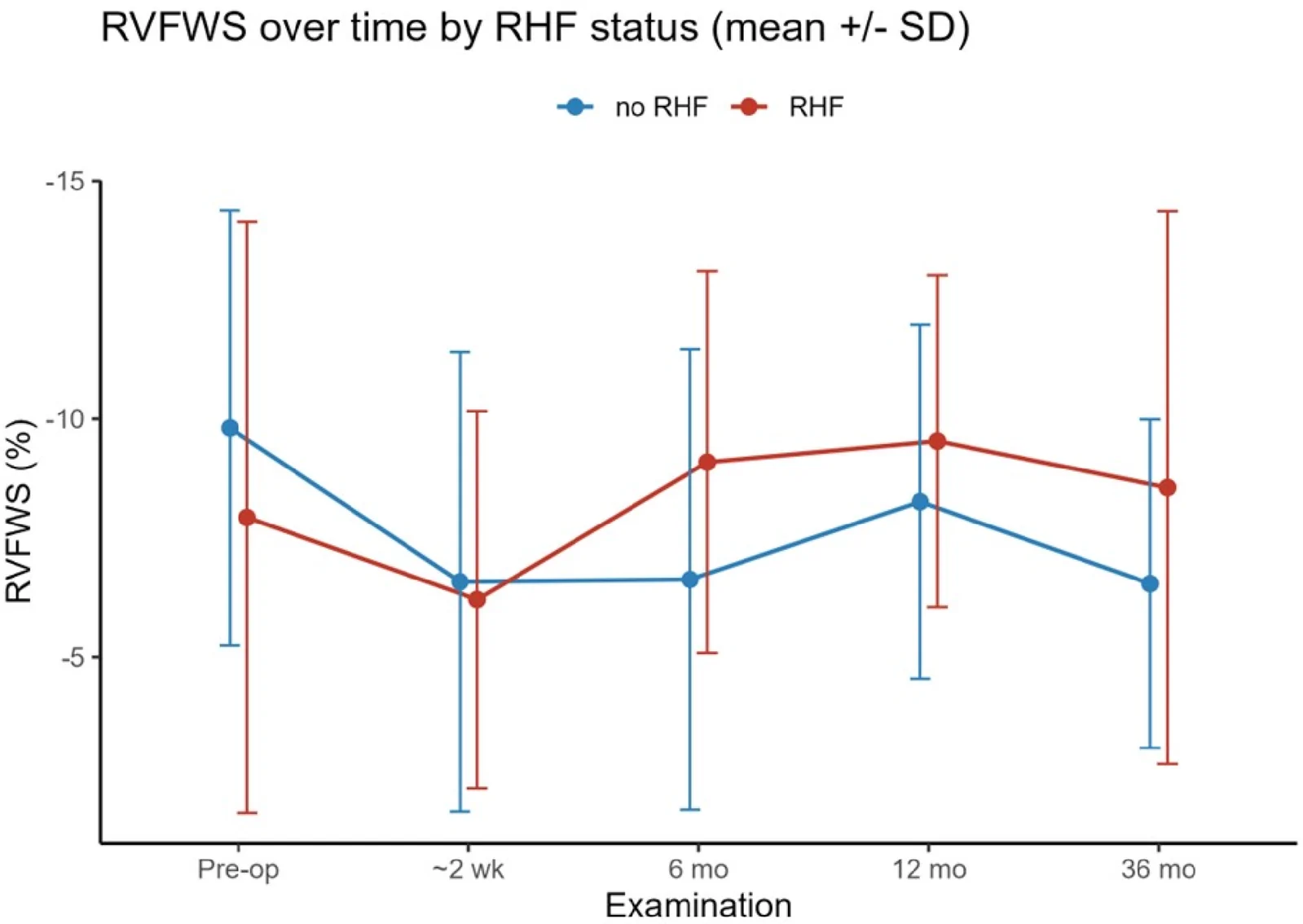
RV free wall strain (RVFWS) at consecutive examinations stratified by RHF status (mean +/- SD)

RVFWS trajectories are shown in Fig. 3; Table 4. In the LMM the group-by-examination interaction approached significance (*p* = 0.081), driven by divergence at 6 months. In patients without RHF, RVFWS declined significantly early after implantation (Δ + 3.3 at 2 weeks, *p* = 0.005; +3.0 at 6 months, *p* = 0.022 relative to baseline) before returning toward baseline. This early decline was replicated in the complete-case model (*n* = 19; 2 weeks, *p* = 0.024) and in the random-slope model (2 weeks, *p* = 0.002; 6 months, *p* = 0.014), confirming its robustness. In the RHF group, no within-group change reached significance. Between-group differences were not significant at any single examination. Full LMM output is provided in Supplementary Tables 1 A-E.

**Table 4:** Right ventricular free-wall strain (RVFWS) during each subsequent examination by RHF status

Exam	Time point	No RHF (*n*)	No RHF	RHF (*n*)	RHF	*p*
1	Pre-op	28	−9.8 ± 4.6	29	−7.9 ± 6.2	0.037
2	~ 2 weeks	27	−6.6 ± 4.8	28	−6.2 ± 4.0	0.913
3	6 months	22	−6.6 ± 4.8	24	−9.1 ± 4.0	0.069
4	12 months	17	−8.3 ± 3.7	14	−9.5 ± 3.5	0.335
5	36 months	12	−6.5 ± 3.4	10	−8.6 ± 5.8	0.350

### RV global longitudinal strain during follow-up

**Fig. 4:**
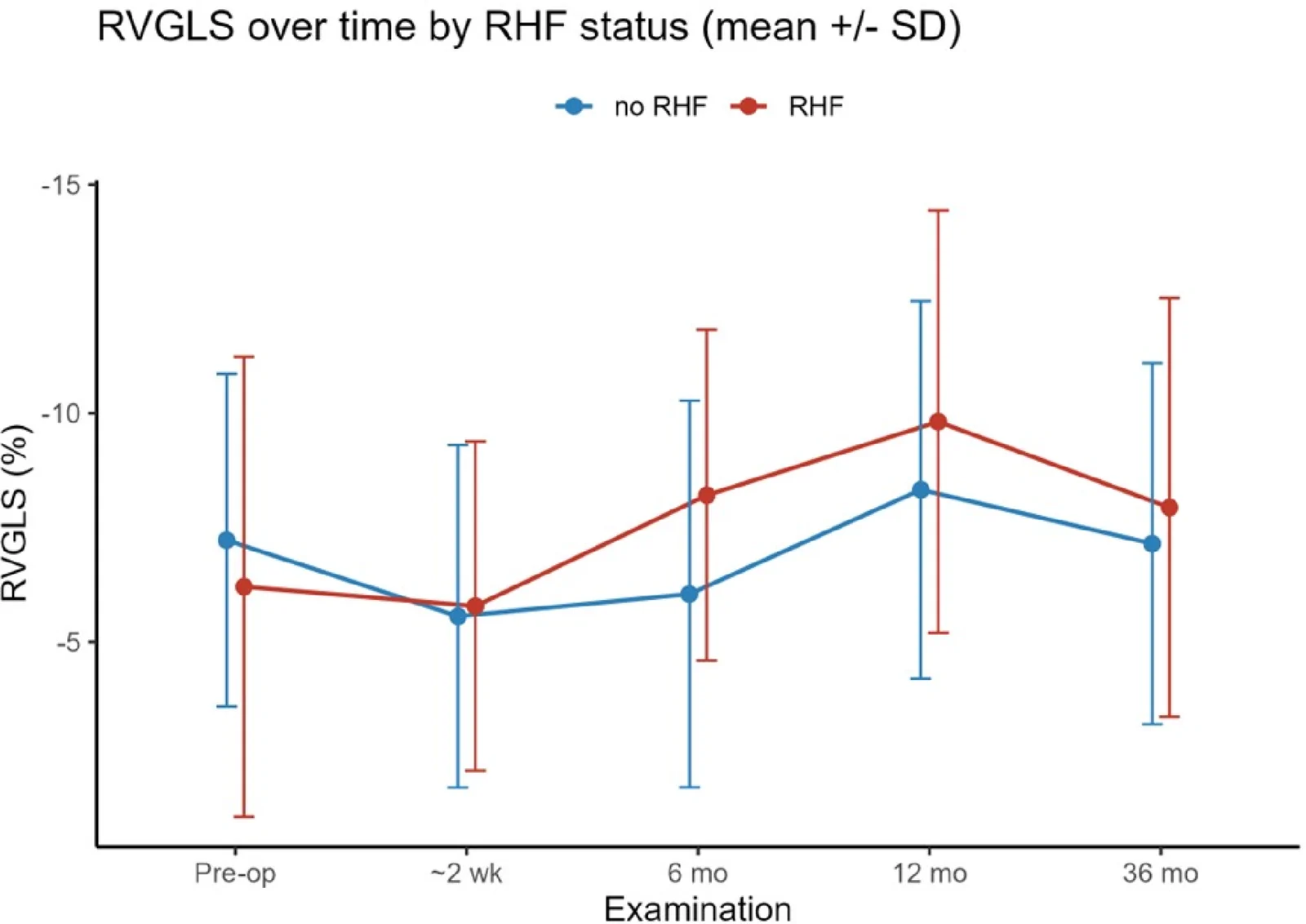
RV global longitudinal strain (RVGLS) at consecutive examinations stratified by RHF status (mean +/- SD)

RVGLS trajectories are shown in Fig. 4; Table 5. The overall group-by-examination interaction was not significant (*p* = 0.213). Within the RHF group, RVGLS improved over time, reaching significance at 12 months (Δ −3.3 vs. baseline, *p* = 0.010; 6 months *p* = 0.074), consistent with RV recovery after temporary support; this improvement was attenuated and non-significant in the smaller complete-case model and should be interpreted with caution. In patients without RHF, RVGLS did not change significantly from baseline. Between-group differences were not significant at any examination (closest at 6 months, *p* = 0.092). Full LLM output is provided in Supplementary Tables 2 A-E.

**Table 5:** Right ventricular global longitudinal strain (RVGLS) during each subsequent examination by RHF status

Exam	Time point	No RHF (*n*)	No RHF	RHF (*n*)	RHF	*p*
1	Pre-op	28	−7.2±3.6	29	−6.2±5.0	0.158
2	~2 weeks	27	−5.6± 3.7	28	−5.8±3.6	0.840
3	6 months	22	−6.0± 4.2	24	−8.2±3.6	0.071
4	12 months	17	−8.3±4.1	14	−9.8±4.6	0.358
5	36 months	12	−7.2±3.9	10	−7.9±4.6	0.673

### Receiver Operating Characteristic (ROC) Analysis

**Fig. 5:**
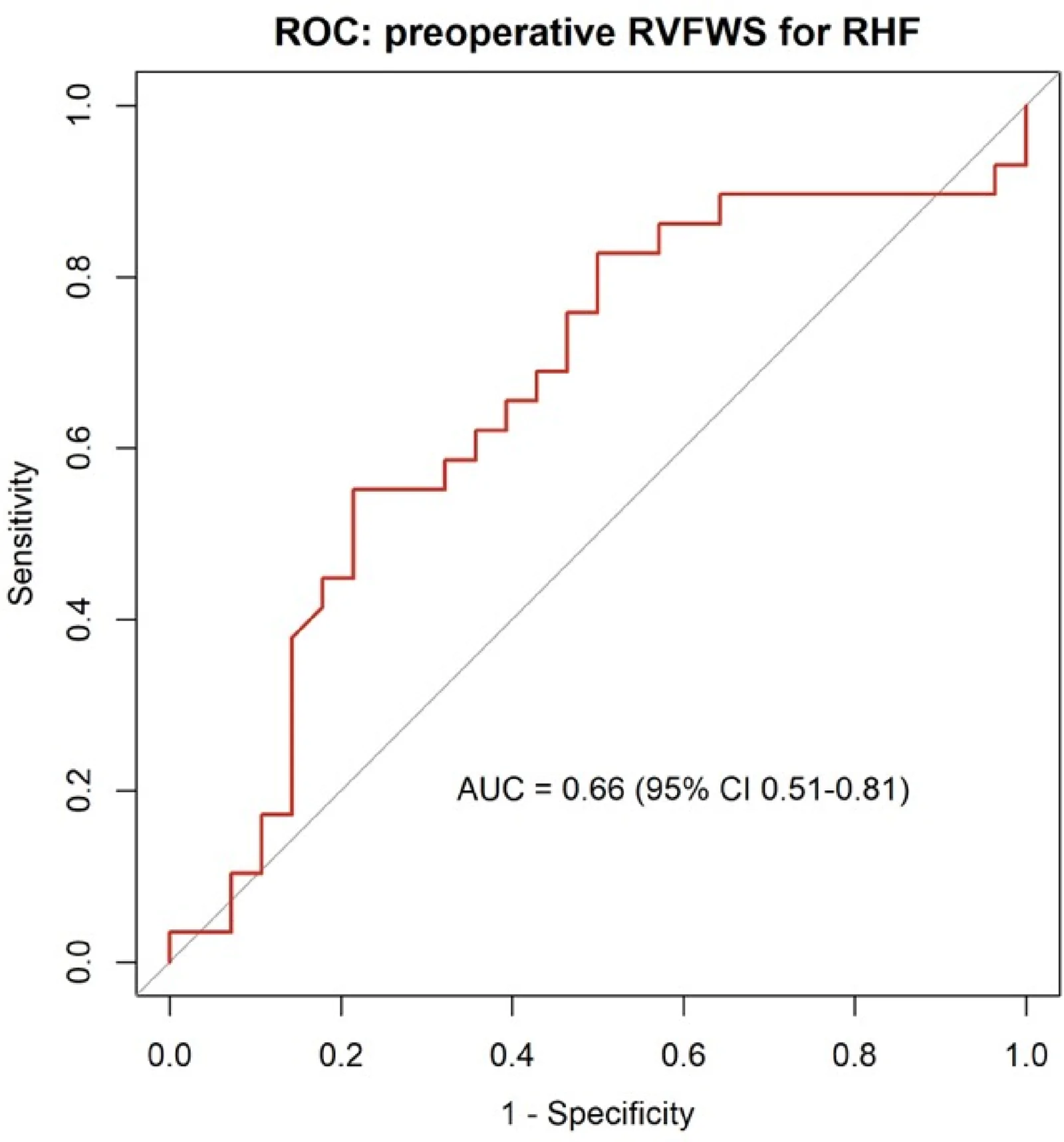
Receiver-Operating-Curve of preoperative RVFWS for prediction of RHF (AUC 0.66, 95% CI 0.52-0.81)

Preoperative RVFWS predicted RHF with an AUC of 0.66 (95% CI 0.52–0.81), an optimal cut-off of −6.3%, 55.2% sensitivity and 78.6% specificity (Fig. 5). Other parameters discriminated poorly (RVGLS 0.61, TAPSE 0.59, FAC 0.66, RVEDD/LVEDD 0.48), and the DeLong test showed no significant difference between RVFWS and the other parameters (all *p* > 0.10). On multivariable logistic regression including RVFWS, TAPSE and RVEDD/LVEDD, no parameter was an independent predictor (RVFWS odds ratio 1.12 per 1% less-negative strain, 95% CI 0.99–1.30, *p* = 0.088).

### Survival Analysis

**Fig. 6:**
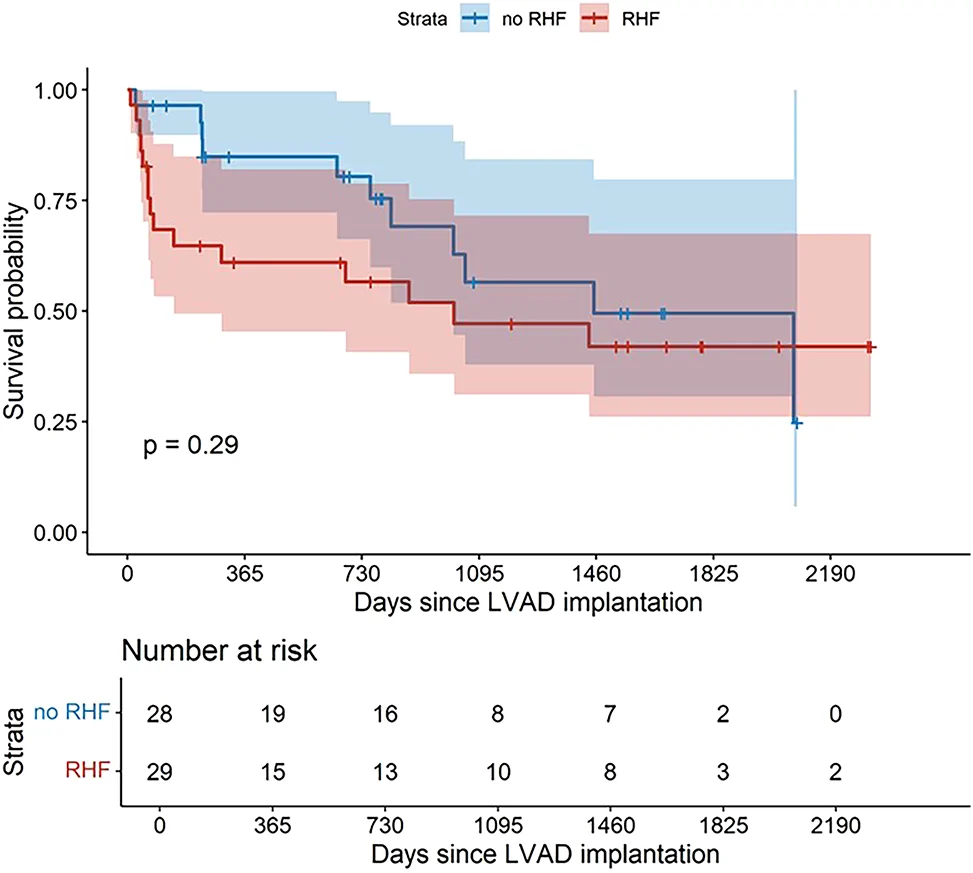
Kaplan-Meier survival by RHF status with numbers at Risk

Survival is shown in Fig. 6. One- and two-year survival rates were 84.9% and 80.4% in the no-RHF group, versus 61.0% and 56.6% in the RHF group. The log-rank test (*p* = 0.293) and the single Cox hazard ratio (1.52, 95% CI 0.70–3.31, *p* = 0.295) were not significant; however, the proportional-hazards assumption was violated (Schoenfeld *p* = 0.025), so these summaries understate a time-varying effect. RMST to 2 years was 169 days shorter in the RHF group (474.8 vs. 643.7 days; difference − 168.9 days, *p* = 0.018). Hazard ratios stratified by time confirmed significantly higher mortality in the RHF group during the first postoperative year (hazard ratio approximately 3.2; equivalently, no-RHF vs. RHF 0.31, 95% CI 0.10–0.97, *p* = 0.044) with no significant difference thereafter. The two-year cumulative incidence of death was 14.3% (no RHF) versus 32.0% (RHF), while the cumulative incidence of transplantation was similar (about 15% in both groups); Gray test *p* = 0.57 and Fine-Gray subdistribution hazard ratio 1.30 (95% CI 0.59–2.84) were concordant in direction but underpowered.

## Discussion

This study evaluates RVFWS and RVGLS in patients before and during 36 months after LVAD implantation. To the best of our knowledge, this is the first study to evaluate serial RV strain parameters after implantation of the HeartMate 3 over a long-term follow-up period.

RHF after LVAD implantation is a very serious clinical problem and concerns 25–40% of patients, depending on the reporting center and the definition applied [15]. We used the updated 2020 MCS-ARC definition, whereas many of the cited studies used the earlier 2012 definition or center-specific criteria, and the timing of RVAD implantation (intraoperative or postoperative) also varied between studies [11, 12, 16]. Because the individual RHF phenotypes were small in number, early acute and early postoperative RHF were combined into a single RHF group for the present analysis; this increased statistical power while remaining consistent with current definitions.

Compared with previously published LVAD cohorts, our patients presented with more advanced heart failure. In this study, 45% of patients in the RHF group were on preoperative MCS (61% in the eaRHF group). Furthermore, a higher proportion of patients were in INTERMACS 1–2, with 48% in INTERMACS 1 in the RHF group. This contrasts with Liang et al. [10], in which fewer than 30% of patients required pre-implant MCS, and only 28% were classed as INTERMACS 1 in the RHF group. These baseline differences suggest a more advanced hemodynamic compromise in our population, which could have influenced the predictive performance of strain-based models.

The combined two-group analysis showed that preoperative RVFWS was significantly less negative in patients who developed RHF, whereas RVGLS, TAPSE, RVEDD/LVEDD and TASV did not differ significantly between groups. Preoperative RVFWS predicted RHF with an area under the curve of 0.66 (95% CI 0.52–0.81), an optimal cut-off of −6.3%, a sensitivity of 55% and a specificity of 79%. These values closely replicate those of Dufendach et al., who reported an optimal RVFWS cut-off of −5.64% with a c-index of 0.65 in 137 patients, with RVGLS again not predictive [17]. Our discrimination was more modest than that of Liang et al., whose four-chamber RV longitudinal strain achieved an AUC of 0.86 in a less severely ill population [10]. The low, less-negative cut-off in our cohort is consistent with the wide range of published RVFWS thresholds (approximately − 5% to −15%) and most likely reflects the uniformly depressed preoperative RV function and limited contractile reserve of a sicker, lower-INTERMACS population [7, 18, 19].

No echocardiographic parameter was an independent predictor of RHF in multivariable analysis, consistent with Grant et al., who found that FAC and TAPSE, were not independent predictors, and that RVFWS added incrementally to the Michigan risk score, increasing its area under the curve from 0.66 to 0.77 [18].

Across the cohort, RVFWS declined early after implantation, reaching statistical significance in the no-RHF group at 2 weeks (*p* = 0.005) and 6 months (*p* = 0.022) before returning toward baseline. Cardiac surgery is known to shift the RV contraction pattern from longitudinal to transverse shortening, thereby reducing measured free-wall strain [20–22]. This early reduction in free-wall deformation may relate to intraoperative ischemia and the use of cardiopulmonary bypass [23].

The trajectory of RVGLS differed from that of RVFWS and is equally informative. Whereas free-wall strain in the RHF group remained largely static, RVGLS in this group improved over time, reaching significance at 12 months. In the early acute phenotype, the temporary RVAD decompresses the RV and mitigates the post-surgical disruption of RV contraction [24–26], and 14 of 18 such patients were still supported at the 2-week examination; the later improvement in RVGLS is consistent with genuine RV recovery once the ventricle is unloaded, mirroring the mid-term recovery of RV systolic function reported with serial strain after LVAD implantation [27], and with progressive accommodation of the increased RV afterload sensitivity that follows left ventricular unloading [28]. This dissociation between a stable free-wall strain and an improving global strain is mechanistically plausible: because RVGLS incorporates the interventricular septum, it reflects the changing septal contribution that follows left ventricular unloading, whereas free-wall strain does not; the two indices therefore describe complementary, not interchangeable, facets of RV adaptation. This improvement should nonetheless be interpreted cautiously, as it was attenuated and non-significant in the smaller complete-case model, and progressive attrition - including transplantation and death - may enrich later time points for survivors with better-recovering ventricles. Critically, absolute strain did not differ between groups at any single examination, suggesting that the informative signal in this cohort lay in serial within-patient change rather than in any cross-sectional measurement.

These longitudinal findings carry potential clinical implications. If serial RV strain reliably tracks RV adaptation, it could be incorporated into structured post-implant surveillance to monitor recovery, inform the timing of weaning of temporary RV support, and flag patients at risk of late right heart failure, which remains an important yet under-recognized cause of morbidity during long-term support [29]. Because within-patient trends were more discriminating than single values, repeated standardized strain assessment rather than a one-off preoperative measurement may prove the more useful monitoring strategy. These hypotheses are exploratory and require prospective validation.

The present outcome data demonstrate a clinically meaningful survival disadvantage for patients with RHF. One- and two-year survival was 85% and 80% in the no-RHF group versus 61% and 57% in the RHF group. RSMT showed a loss of 169 days over two years in the RHF group (*p* = 0.018), and time-stratified analysis localized the excess mortality to the first postoperative year (hazard ratio approximately 3.2; *p* = 0.044), with no significant difference thereafter. This pattern of an early hazard that attenuates over time is physiologically plausible for perioperative RV failure and reconciles the visibly separated survival curves with the non-significant log-rank result. Heart transplantation occurred at similar rates in both groups and competing-risks analysis was concordant, so the mortality difference is not explained by differential transplantation. These findings are consistent with registry data [3, 29] indicating that early and severe RHF carries substantial prognostic weight, whereas transient postoperative RHF may resolve with medical or temporary mechanical support.

Several limitations apply. The single-center, retrospective design introduces selection bias, and the exclusion of 38 of 95 patients for inadequate image quality, to which RV strain is particularly sensitive, may limit generalisability. The analytic cohort is small with progressive attrition during follow-up; for the principal strain comparison, only a difference of approximately 3.7 strain points would have been detectable at 80% power, so non-significant between-group contrasts should be regarded as underpowered rather than truly negative. Mixed models used all available observations under a missing-at-random assumption, and complete-case analyses were directionally consistent for RVFWS but attenuated for the RVGLS recovery signal. Inter- and intra-observer reproducibility was not formally assessed, although all measurements were obtained on a single validated vendor platform by experienced operators. Finally, in some patients, the preoperative echocardiogram was obtained during temporary MCS, which may have influenced baseline strain values.

## Conclusion

In this retrospective single-centre cohort, preoperative RVFWS showed only modest discrimination for right heart failure after HeartMate 3 implantation, whereas no other echocardiographic parameter and no multivariable combination improved prediction. Longitudinal modelling revealed dynamic changes in RV mechanics that single measurements did not capture: an early postoperative decline in RVFWS n in patients without RHF and a later recovery of RVGLS in those with RHF. RHF was associated with a clinically meaningful survival disadvantage concentrated in the first postoperative year and was demonstrable in time-appropriate analyses despite a non-significant log-rank test. Taken together, serial RV strain may complement established preoperative risk assessment, but larger prospective studies with standardized follow-up are needed to define prognostic thresholds and clinical utility.

## Supplementary Information

Below is the link to the electronic supplementary material.

Supplementary Material 1

## Data Availability

All data used to create this manuscript will be made available upon request.
