# Supplementary material for: Assessment of the predictive value of right ventricular longitudinal strain and functional trends in right ventricular function of patients after LVAD implantation
Source: Int J Cardiovasc Imaging. 2026 Sep 17;42(11):2135–45. doi: 10.1007/s10554-026-03780-z (PMC13745418; doi:10.1007/s10554-026-03780-z)
Supplement: Supplementary file 1 [file 10554_2026_3780_MOESM1_ESM.docx]

Supplementary Tables

**Supplementary Tables 1A-E.** Full linear mixed-model output for RV free wall strain (RVFWS) (group x examination, random intercept per patient; REML, Satterthwaite degrees of freedom, Tukey-adjusted contrasts).

**A. Fixed effects (type III F-tests)**

| **Effect** | **NumDF** | **DenDF** | **F** | **p** |
| --- | --- | --- | --- | --- |
| Group | 1 | 58.8 | 0.06 | 0.804 |
| Examination | 4 | 152.7 | 4.20 | 0.003 |
| Group x examination | 4 | 152.7 | 2.12 | 0.081 |

**B. Fixed-effect coefficients**

| **Term** | **Estimate** | **95% CI** | **p** |
| --- | --- | --- | --- |
| Intercept (no RHF, pre-op) | -9.81 | -11.55, -8.06 | <0.001 |
| RHF (vs no RHF) | 1.88 | -0.57, 4.32 | 0.132 |
| 2 weeks (vs pre-op) | 3.26 | 1.31, 5.21 | 0.001 |
| 6 months | 2.97 | 0.89, 5.05 | 0.005 |
| 12 months | 1.04 | -1.24, 3.31 | 0.370 |
| 36 months | 3.31 | 0.72, 5.90 | 0.013 |
| RHF x 2 weeks | -1.52 | -4.25, 1.21 | 0.273 |
| RHF x 6 months | -4.16 | -7.05, -1.26 | 0.005 |
| RHF x 12 months | -2.03 | -5.37, 1.30 | 0.230 |
| RHF x 36 months | -2.88 | -6.67, 0.92 | 0.136 |

**C. Estimated marginal means, strain % (95% CI)**

| **Examination** | **No RHF** | **RHF** |
| --- | --- | --- |
| Pre-op | -9.81 (-11.55, -8.06) | -7.93 (-9.65, -6.22) |
| 2 weeks | -6.55 (-8.32, -4.78) | -6.19 (-7.93, -4.46) |
| 6 months | -6.84 (-8.75, -4.92) | -9.12 (-10.96, -7.27) |
| 12 months | -8.77 (-10.90, -6.65) | -8.93 (-11.23, -6.63) |
| 36 months | -6.50 (-8.96, -4.05) | -7.50 (-10.16, -4.84) |

**D. Within-group change from baseline, Delta (Tukey p)**

| **Contrast** | **No RHF: Delta (p)** | **RHF: Delta (p)** |
| --- | --- | --- |
| 2 weeks vs pre-op | +3.26 (0.005) | +1.74 (0.267) |
| 6 months vs pre-op | +2.97 (0.022) | -1.18 (0.680) |
| 12 months vs pre-op | +1.04 (0.843) | -1.00 (0.888) |
| 36 months vs pre-op | +3.31 (0.050) | +0.43 (0.997) |

**E. Between-group difference at each examination (no RHF - RHF)**

| **Examination** | **Delta (no RHF - RHF)** | **p** |
| --- | --- | --- |
| Pre-op | -1.88 | 0.132 |
| 2 weeks | -0.35 | 0.778 |
| 6 months | +2.28 | 0.093 |
| 12 months | +0.16 | 0.922 |
| 36 months | +1.00 | 0.587 |

**Abbreviations/notes:** random-intercept SD 2.92, residual SD 3.65; adjusted intraclass correlation 0.39. A random-slope model fitted the data better than the random-intercept model (likelihood-ratio chi-square 12.99, df 2, p=0.002); the significant early decline in RVFWS in the no-RHF group persisted in the random-slope and complete-case (n=19) sensitivity analyses. Delta values are estimated marginal mean differences (strain %). Positive Delta indicates less-negative (worse) strain.

**Supplementary Tables 2A-E.**

Full linear mixed-model output for RV global longitudinal strain (RVGLS) (group x examination, random intercept per patient; REML, Satterthwaite degrees of freedom, Tukey-adjusted contrasts).

**A. Fixed effects (type III F-tests)**

| **Effect** | **NumDF** | **DenDF** | **F** | **p** |
| --- | --- | --- | --- | --- |
| Group | 1 | 60.2 | 0.32 | 0.573 |
| Examination | 4 | 153.7 | 5.55 | <0.001 |
| Group x examination | 4 | 153.7 | 1.47 | 0.213 |

**B. Fixed-effect coefficients**

| **Term** | **Estimate** | **95% CI** | **p** |
| --- | --- | --- | --- |
| Intercept (no RHF, pre-op) | -7.23 | -8.75, -5.70 | <0.001 |
| RHF (vs no RHF) | 1.01 | -1.12, 3.15 | 0.350 |
| 2 weeks (vs pre-op) | 1.70 | 0.02, 3.37 | 0.048 |
| 6 months | 0.93 | -0.87, 2.72 | 0.310 |
| 12 months | -1.36 | -3.32, 0.61 | 0.174 |
| 36 months | 0.25 | -1.98, 2.48 | 0.824 |
| RHF x 2 weeks | -1.26 | -3.61, 1.10 | 0.293 |
| RHF x 6 months | -3.01 | -5.51, -0.51 | 0.018 |
| RHF x 12 months | -1.92 | -4.80, 0.95 | 0.188 |
| RHF x 36 months | -1.30 | -4.57, 1.97 | 0.434 |

**C. Estimated marginal means, strain % (95% CI)**

| **Examination** | **No RHF** | **RHF** |
| --- | --- | --- |
| Pre-op | -7.23 (-8.75, -5.70) | -6.21 (-7.71, -4.71) |
| 2 weeks | -5.53 (-7.08, -3.98) | -5.77 (-7.29, -4.25) |
| 6 months | -6.30 (-7.97, -4.63) | -8.29 (-9.91, -6.68) |
| 12 months | -8.58 (-10.43, -6.73) | -9.49 (-11.49, -7.49) |
| 36 months | -6.97 (-9.11, -4.84) | -7.26 (-9.56, -4.95) |

**D. Within-group change from baseline, Delta (Tukey p)**

| **Contrast** | **No RHF: Delta (p)** | **RHF: Delta (p)** |
| --- | --- | --- |
| 2 weeks vs pre-op | +1.70 (0.178) | +0.44 (0.974) |
| 6 months vs pre-op | +0.93 (0.773) | -2.08 (0.074) |
| 12 months vs pre-op | -1.36 (0.535) | -3.28 (0.010) |
| 36 months vs pre-op | +0.25 (0.999) | -1.05 (0.861) |

**E. Between-group difference at each examination (no RHF - RHF)**

| **Examination** | **Delta (no RHF - RHF)** | **p** |
| --- | --- | --- |
| Pre-op | -1.01 | 0.350 |
| 2 weeks | +0.24 | 0.825 |
| 6 months | +1.99 | 0.092 |
| 12 months | +0.91 | 0.512 |
| 36 months | +0.28 | 0.859 |

**Abbreviations/notes:** random-intercept SD 2.61, residual SD 3.14; adjusted intraclass correlation 0.41. A random-slope model did not improve fit (likelihood-ratio chi-square 3.27, df 2, p=0.195), so a random intercept was retained. The within-group improvement of RVGLS in the RHF group at 12 months (p=0.010) was attenuated and non-significant in the complete-case (n=19) sensitivity analysis. Delta values are estimated marginal mean differences (strain %). Negative Delta indicates more-negative (better) strain.

**Supplementary Figure S1**

Diagnostic plots for the RV free wall strain (RVFWS) linear mixed-effects model. Left: residuals versus fitted values; centre: quantile-quantile plot of the model residuals; right: quantile-quantile plot of the patient-level random intercepts. Residuals are approximately symmetric about zero and the quantile-quantile plots are near-linear apart from a few low-end outliers, supporting the normality and homoscedasticity assumptions.

**Supplementary Figure S2**

Diagnostic plots for the RV global longitudinal strain (RVGLS) linear mixed-effects model. Panels as in Supplementary Figure S1. Residual and random-intercept distributions are consistent with model assumptions, with minor tail deviations.
